# American Medical Association

**Published:** 1873-05-15

**Authors:** 


MEDICAL EXAMINER.

*A Semi-Monthly Journal of Medical Sciences.*

EDITED BY

N. S. DAVIS, M. D., AND F. H. DAVIS, M. D.

Chicago, May 15th, 1873.

### EDITORIAL.

### AMERICAN MEDICAL ASSOCIATION.

The annual meeting of this great National organization commenced in St. Louis, Mo., on the morning of Tuesday, May 6th, 1873, and was closed at noon on the Friday following. Not less than 450 members were in attendance, embracing representatives from thirty-two States and Territories. The reception and treatment of the members of the Association by the Committee of Arrangements, the profession and citizens of St. Louis, and the daily press, was cordial, dignified, and honorable. The press especially is worthy of commendation for having indulged in none of that spirit of facetiousness and sensationalism which has so often of late years characterized the reports in the papers of other cities. The business of the general morning sessions was transacted in order, and with a spirit of harmony and good feeling seldom witnessed in so large an assembly.

As has so often happened in previous years, there had not been as complete provision made for the accommodation of all the *Sections* as is desirable; and the number of papers and reports to be referred was less than usual. Yet several of the Sections were well attended, and their time fully and profitably occupied. As we shall give an abstract of the proceedings in our next number, further comments are deferred for the present.

